# MRI-based endplate bone quality predicts bony fusion and cage subsidence after PLIF in patients with Modic changes

**DOI:** 10.3389/fphys.2026.1925075

**Published:** 2026-09-17

**Authors:** Xiaoxia Qiao, Zhao Guo, Tong Li, Jie Zhang, Xiong Zhang, Xiaozhe Zhou

**Affiliations:** Affiliated Hospital of Hebei University, Baoding, China

**Keywords:** bony fusion, cage subsidence, endplate bone quality, endplate sclerosis, Modic changes, posterior lumbar interbody fusion

## Abstract

**Background:**

Modic changes (MCs) are classified into non- sclerotic and sclerotic types based on endplate morphology. Our previous work showed that non- sclerotic MCs increase cage subsidence risk after posterior lumbar interbody fusion (PLIF), while sclerotic MCs reduce bony fusion rates. Endplate bone quality (EBQ), an magnetic resonance imaging (MRI)- derived surrogate for regional bone density, may predict these distinct complications. This study evaluated EBQ’s predictive value for cage subsidence in the non- sclerotic group and for bony fusion failure in the sclerotic group.

**Methods:**

We retrospectively reviewed 189 patients (mean age 52.6 years, 62.5% female) who underwent single- segment PLIF for degenerative lumbar disease with MCs. Patients were divided into non- sclerotic (n=108) and sclerotic (n=81) groups based on preoperative computed tomography (CT) and MRI. EBQ was calculated as the average signal intensity of the subchondral bone 3 mm above and below the surgical level divided by the cerebrospinal fluid signal at L3. Cage subsidence and bony fusion were assessed on CT at minimum 1- year follow- up. Binary logistic regression and receiver operating characteristic (ROC) curve analyses were performed.

**Results:**

In the non- sclerotic group, higher EBQ independently predicted cage subsidence (OR = 1.539, 95% CI: 1.034- 2.291, P<0.05), with an AUC of 0.809 (95% CI: 0.684- 0.934). The optimal cutoff was 3.015, yielding 90.0% sensitivity and 67.3% specificity. In the sclerotic group, lower EBQ independently predicted non- fusion (OR = 1.404, 95% CI: 1.007- 1.956, P<0.05), with an AUC of 0.784 (95% CI: 0.675- 0.892) and optimal cutoff of 1.745 (72.6% sensitivity, 78.9% specificity).

**Conclusion:**

EBQ serves as a dual predictive biomarker: high EBQ identifies non- sclerotic patients at risk for cage subsidence, while low EBQ identifies sclerotic patients at risk for fusion failure. Preoperative EBQ assessment can guide personalized surgical strategies to reduce complications and improve outcomes.

## Introduction

Modic changes (MCs) refer to bone marrow and endplate lesions adjacent to degenerative lumbar discs, characterized by abnormal MRI signals in the endplates and subchondral bone (Modic et al., 1988b; Modic et al., 1988a). Notably, MCs are not associated with malignant bone marrow neoplasms, spinal infections, or seropositive rheumatic diseases (Jensen et al., 2008). The overall prevalence of MCs is approximately 5.8% (range: 1.6%- 13.3%) in patients with low back pain and degenerative disc disease (Modic et al., 1988b). Furthermore, MCs are more commonly observed in patients undergoing long- term glucocorticoid therapy and in those with poor posture (Mok et al., 2016; Kanna et al., 2017). The cartilaginous endplate serves as the primary pathway for nutrient transport to the intervertebral disc, and its morphology and thickness are critical for regulating nutrient supply. Endplate degeneration in MCs accelerates disc degeneration, thereby driving the progression of degenerative lumbar spinal disorders. Consequently, many affected patients eventually require PLIF surgery (Kim et al., 2018).

In our previous work, we classified MCs into non-sclerotic and sclerotic groups based on endplate integrity. The non-sclerotic type is associated with an increased risk of cage subsidence after PLIF, though without a significant impact on clinical outcomes. In contrast, the sclerotic type can compromise fusion rates, which subsequently leads to poorer long-term clinical outcomes as reflected in elevated VAS and ODI scores (Zhang et al., 2025). We hypothesize that these distinct outcomes stem from differences in endplate bone mineral density (BMD) between the two groups.

The EBQ score, derived from MRI signal intensity of subchondral bone relative to cerebrospinal fluid, does not directly measure BMD (e.g., by DEXA) but rather integrates density and tissue composition information derived from T1-weighted MRI signal intensity (Jones et al., 2021; Ran et al., 2024; Zheng et al., 2024). In non- sclerotic endplates, lower EBQ (i.e., lower density) indicates reduced mechanical competence of the subchondral bone, making it susceptible to collapse under the compressive loads of an interbody cage. Conversely, in sclerotic endplates, higher EBQ (i.e., higher density) paradoxically signifies an avascular, sclerotic scar tissue with reduced osteogenic potential and impaired exchange of nutrients and growth factors across the endplate. This biological environment hinders the recruitment of osteoprogenitor cells from the vertebral body marrow and delays the incorporation of bone graft, ultimately increasing non- union rates (Xiao et al., 2023; Zeng et al., 2023; Fan et al., 2024). Thus, EBQ serves as a dual marker: in low- density bone it predicts mechanical failure (subsidence), while in high- density sclerotic bone it predicts biological failure (non- fusion). Prior studies have established EBQ’s independent predictive value for prosthesis subsidence following transforaminal lumbar interbody fusion (TLIF) (Ai et al., 2024). However, its utility in the context of MCs has not been explored.

This study aims to investigate the imaging findings and clinical outcomes of PLIF surgery in patients with MCs, utilizing the EBQ score to predict postoperative fusion in the sclerotic group and cage subsidence in the non-sclerotic group, respectively.

## Methods

### Patient population

A retrospective study was conducted on patients undergoing PLIF at our institution between October 2017 and August 2022. Ethical approval was secured from the Institutional Review Board. Informed consent was waived by the ethics committee because this was a retrospective analysis of de- identified clinical data.

Patients were selected based on specific inclusion and exclusion criteria. Inclusion criteria were: (1) Age ≥ 18 years; (2) single-segment PLIF; (3) Voluntary acceptance of surgery with signed informed consent. The exclusion criteria included: (1) Diagnosis of spinal tumors, spinal tuberculosis or spinal infection; (2) History of prior lumbar spine surgery; (3) Osteoporosis defined as T- score ≤ -2.5 on dual- energy X- ray absorptiometry (DXA) (WHO criteria); (4) Psychiatric disorders or depression leading to non- compliance with treatment; (5) Incomplete follow- up data.

Data collected from medical records included Age, Sex, Lumbar lordosis, BMI, Spondylolisthesis, Hypertension, and Diabetes.

### Imaging assessment

All preoperative imaging (lumbar X- ray, CT, and MRI) was performed within 2 weeks prior to surgery as part of routine clinical evaluation. Lumbar X- rays (standing anteroposterior and lateral views) were used to measure lumbar lordosis (LL) and to identify spondylolisthesis. MRI (T1- weighted and T2- weighted sequences) was utilized to diagnose lumbar degenerative disease, evaluate Modic changes, and calculate EBQ and VBQ scores. CT (with 1- mm slice thickness and sagittal reconstruction) was used to assess endplate sclerosis for patient grouping and, at the 1- year postoperative follow- up, to evaluate cage subsidence and bony fusion.

All CT and MRI scans were acquired on the same scanners with standardized protocols (see imaging acquisition details below) to minimize inter- modality variability. The CT- based sclerosis classification and the MRI- based EBQ measurement are complementary rather than interchangeable—CT directly visualizes osseous morphology, while MRI reflects marrow composition and bone quality. We did not attempt to directly equate EBQ to CT- based HU values.

Initially, 1561 patients were screened, with 713 excluded according to the predefined inclusion and exclusion criteria. Subsequently, 848 eligible patients were identified, among whom 189 exhibited MCs on MRI. Finally, Sagittal reconstructed CT images were used to identify endplate sclerosis (Liu et al., 2020). The diagnosis of endplate sclerosis was based on the following criteria: (1) clear thickening of the subchondral bone with increased attenuation on CT, measured by region- of- interest (ROI) placed on the affected endplate; a mean HU value > 350 was considered definitive sclerosis; (2) for cases with HU between 300 and 350 (ambiguous), the HU ratio was calculated as the HU of the affected endplate divided by the HU of a normal endplate (L1 or L2, without MCs). A ratio > 1.2 was considered sclerosis; (3) all assessments were performed independently by two spine surgeons; disagreements were resolved by consensus with a third senior musculoskeletal radiologist. Using these criteria, we identified 108 patients without endplate sclerosis and 81 with endplate sclerosis (**Figure 1**).

**Figure 1:**
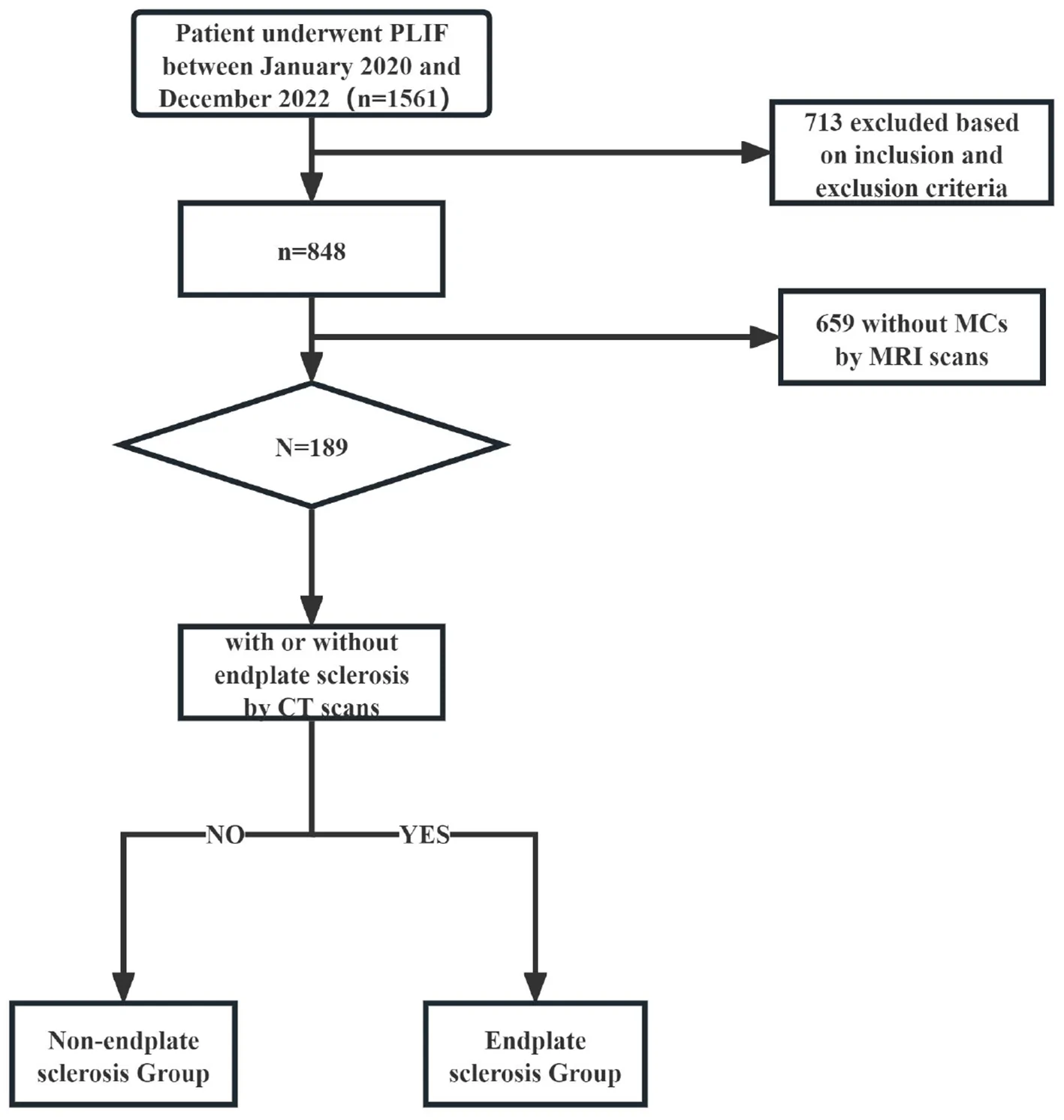
The flow diagram of including patients.

Interobserver and intraobserver ICCs for the diagnosis of sclerosis were 0.885 and 0.861, respectively. (Note: HU value refers to the CT attenuation value.).

### Data collection

We calculated the novel VBQ score for each patient by dividing the median signal intensity of L1–4 vertebrae by the signal intensity of Cerebrospinal Fluid (CSF) (Ehresman et al., 2020).


$$VBQ_{score}\;=\;\frac{SI_{L1-4}}{SI_{CSF}}$$

EBQ was measured by analyzing the subchondral bone 3 mm above and below the surgical level. The average signal intensity of the two endplates was divided by the signal intensity of the CSF at the L3 level (**Figure 2**) (Jones et al., 2022).

**Figure 2:**
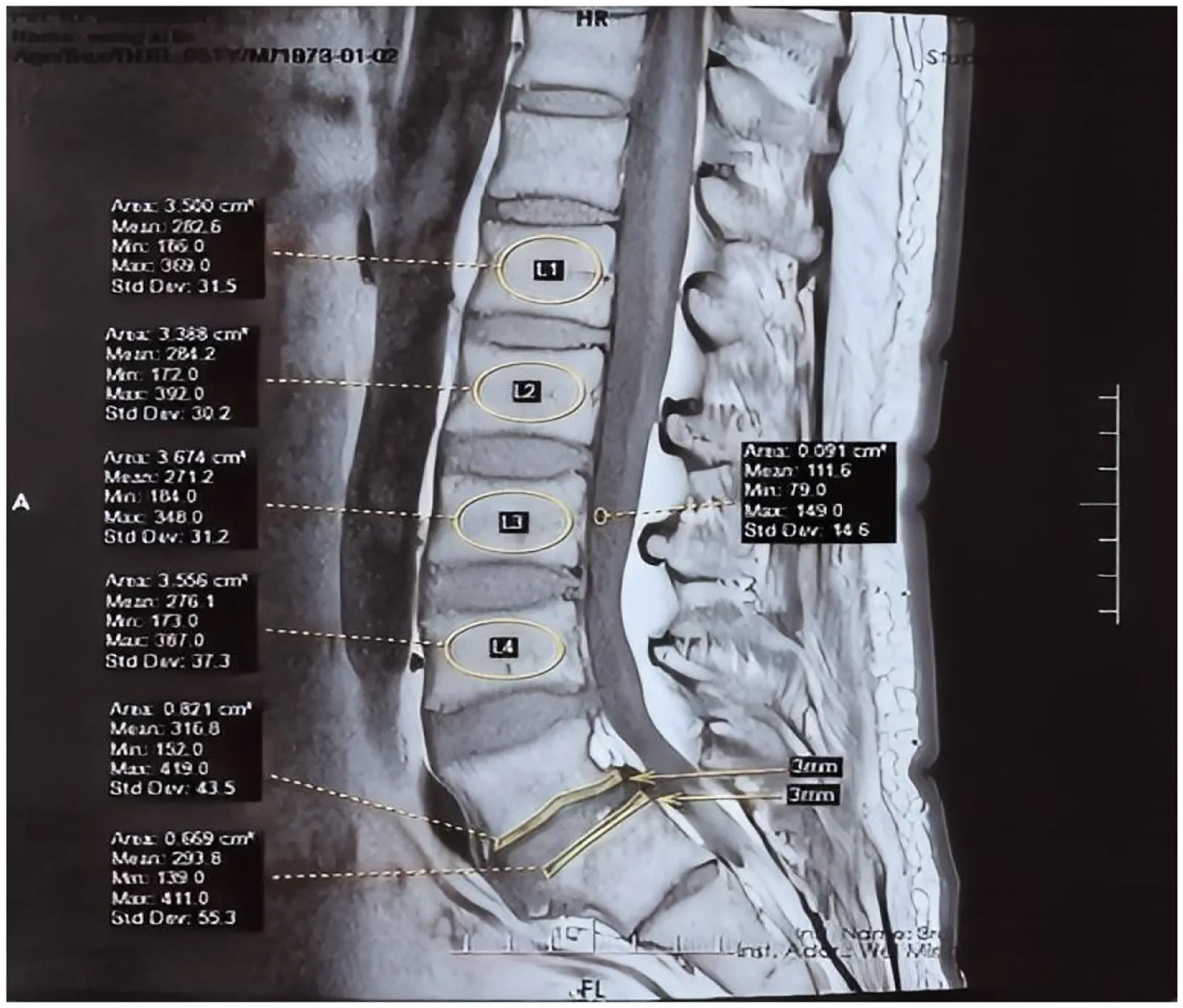
Representative image of ROI used to calculate the VBQ score and EBQ score.


$$EBQ_{score}\;=\;\frac{SI_{targeted\;endplates}}{SI_{CSF}}$$

### Outcome measures

At the one-year postoperative follow-up, cage subsidence and bony fusion were assessed by CT. Cage subsidence was classified according to the Marchi criteria: Grade 0 (0–24% disc height loss), Grade I (25–49%), Grade II (50–74%), and Grade III (75–100%). For analysis, Grades 0 and I were categorized as low-grade subsidence, and Grades II and III as high-grade subsidence. Bony fusion was evaluated using the Brantigan and Steffee criteria, which include five grades: Grade I (definite pseudarthrosis), Grade II (probable pseudarthrosis), Grade III (uncertain), Grade IV (probable fusion), and Grade V (definite fusion). Fusion was defined as Grades IV or V, while Grades I–III indicated non-fusion.

Binary logistic regression was employed to identify predictors of fusion in the sclerotic group and subsidence in the non-sclerotic group.

All procedures were performed under general anesthesia by one of two senior spine surgeons. Two orthopedic surgeons independently evaluated MCs, endplate sclerosis, bony fusion, and cage subsidence to calculate interobserver reliability. Intraobserver reliability was assessed by one surgeon repeating the measurements after a one-week interval. The interobserver and intraobserver intraclass correlation coefficients (ICCs) were 0.885 and 0.861, respectively.

### Statistical analysis

Descriptive statistics are presented as mean ± standard deviation for continuous variables and as frequency (percentage) for categorical variables. Between-group comparisons were performed using independent t- tests for continuous variables and chi-square tests for categorical variables, respectively. Binary logistic regression was used to identify independent predictors, with model fit assessed by the Hosmer- Lemeshow goodness- of- fit test. To evaluate the predictive performance of EBQ, receiver operating characteristic (ROC) curves were generated and the AUC was calculated. The optimal EBQ cut- off value was determined using the Youden index (maximizing sensitivity + specificity – 1). All analyses were conducted using SPSS software, with a p- value < 0.05 considered statistically significant.

## Results

This study included 189 patients (62.5% female), with a mean age of 52.60 years (range: 22–78 years). Among the cohort, 38 patients (25%) were diagnosed with spondylolisthesis, 56 patients (37%) had hypertension, and 38 patients (25%) were identified as having diabetes mellitus.

The patients were categorized into two groups: 108 patients (57.1%) in non-sclerotic group and 81 patients (42.9%) in sclerotic group. Patient demographics are detailed in **Table 1**. No significant differences were observed between the two groups regarding age (p = 0.691), BMI (p = 0.557), sex (p = 0.795), LL (p = 0.121), hypertension (p = 0.127), and diabetes (p = 0.643). Statistically significant differences were found in spondylolisthesis(p <0.01), fusion(p <0.01), cage subsidence(p <0.01) and EBQ (p = 0.036) (**Table 1**).

**Table 1:** Demographic and baseline characteristics of the patients.

Parameters	Total	Non-sclerotic group	Sclerotic group	P
Number	189	108 (57.1%)	81 (42.9%)	
Age (years)	52.44 ± 11.53	52.81 ± 10.99	51.95 ± 12.26	0.691
Sex, (n%)				0.795
Male, (n%)	71 (37.6%)	41 (38%)	30 (37%)	
Female, (n%)	118 (62.5%)	67 (62%)	51 (63%)	
BMI	26.17 ± 4.12	26.29 ± 4.35	26.02 ± 3.82	0.557
Lumbar lordosis	25.71 ± 12.61	25.93 ± 11.19	25.42 ± 14.35	0.121
Spondylolisthesis, (n%)	50 (26.5%)	23 (21.3%)	27 (33.3%)	<0.01
Hypertension, (n%)	73 (38.6%)	39 (36.1%)	34 (42%)	0.127
Diabetes, (n%)	15 (7.9%)	9 (8.3%)	6 (7.4%)	0.643
EBQ	2.48 ± 0.78	2.92 ± 0.66	1.89 ± 0.50	0.036
VBQ	2.79 ± 0.58	2.87 ± 0.58	2.68 ± 0.57	0.57
Cage subsidence	12 (6.3%)	10 (9.3%)	2 (2.5%)	<0.01
Bony fusion	166 (87.9%)	104 (96.3%)	62 (76.5%)	<0.01

Binary logistic regression was performed to analyze various risk factors, including Age, Sex, BMI, Hypertension, Spondylolisthesis, LL, VBQ, and EBQ. The analysis demonstrated that EBQ was an independent risk factor for both cage subsidence and interbody fusion following PLIF in patients with MCs (**Tables 2**, **3**).

**Table 2:** Analysis of risk factors affecting cage subsidence in non-sclerotic group.

Variable	β	S.E.	Wald	P	Exp(B)	95%CI	
Sex	-0.651	1.360	0.229	0.632	0.522	0.036	7.499
Age	0.072	0.047	2.036	0.123	1.075	0.981	1.179
Spondylolisthesis	-1.467	1.380	1.131	0.288	0.231	0.015	3.445
EBQ	0.431	0.203	4.512	0.034	1.539	1.034	2.291
VBQ	1.577	1.189	1.757	0.185	4.839	0.470	49.790
Lumbar lordosis	0.039	0.036	1.149	0.284	1.040	0.968	1.116
BMI	-0.367	0.237	2.399	0.121	0.693	0.435	1.102
Hypertension	-1.052	1.357	0.601	0.438	0.349	0.024	5.088

**Table 3:** Analysis of risk factors affecting bony fusion in sclerotic group.

Variable	β	S.E.	Wald	P	Exp(B)	95%CI	
Sex	-0.073	0.780	0.009	0.926	0.930	0.102	4.289
Age	0.000	0.035	0.000	0.995	1.000	0.933	1.071
Spondylolisthesis	2.487	1.239	3.804	0.045	12.022	1.060	136.390
EBQ	0.339	0.170	3.967	0.046	1.404	1.007	1.956
VBQ	1.249	0.787	2.504	0.114	3.486	0.742	16.366
Lumbar lordosis	-0.028	0.027	1.115	0.291	0.972	0.922	1.025
BMI	0.167	0.131	1.644	0.200	1.182	0.915	1.527
Hypertension	0.781	1.116	0.490	0.484	2.184	0.245	19.456

In the non-sclerotic group, a higher score was significantly associated with an increased risk of interbody cage subsidence [odds ratio (OR) = 1.539; 95% confidence interval (CI): 1.034-2.291; P < 0.05]. ROC curve, AUC for the EBQ score was 0.809 (95% CI: 0.684-0.934).

The optimal EBQ score cutoff was 3.015, yielding a sensitivity of 90.0% and a specificity of 67.3% (**Figure 3**).

**Figure 3:**
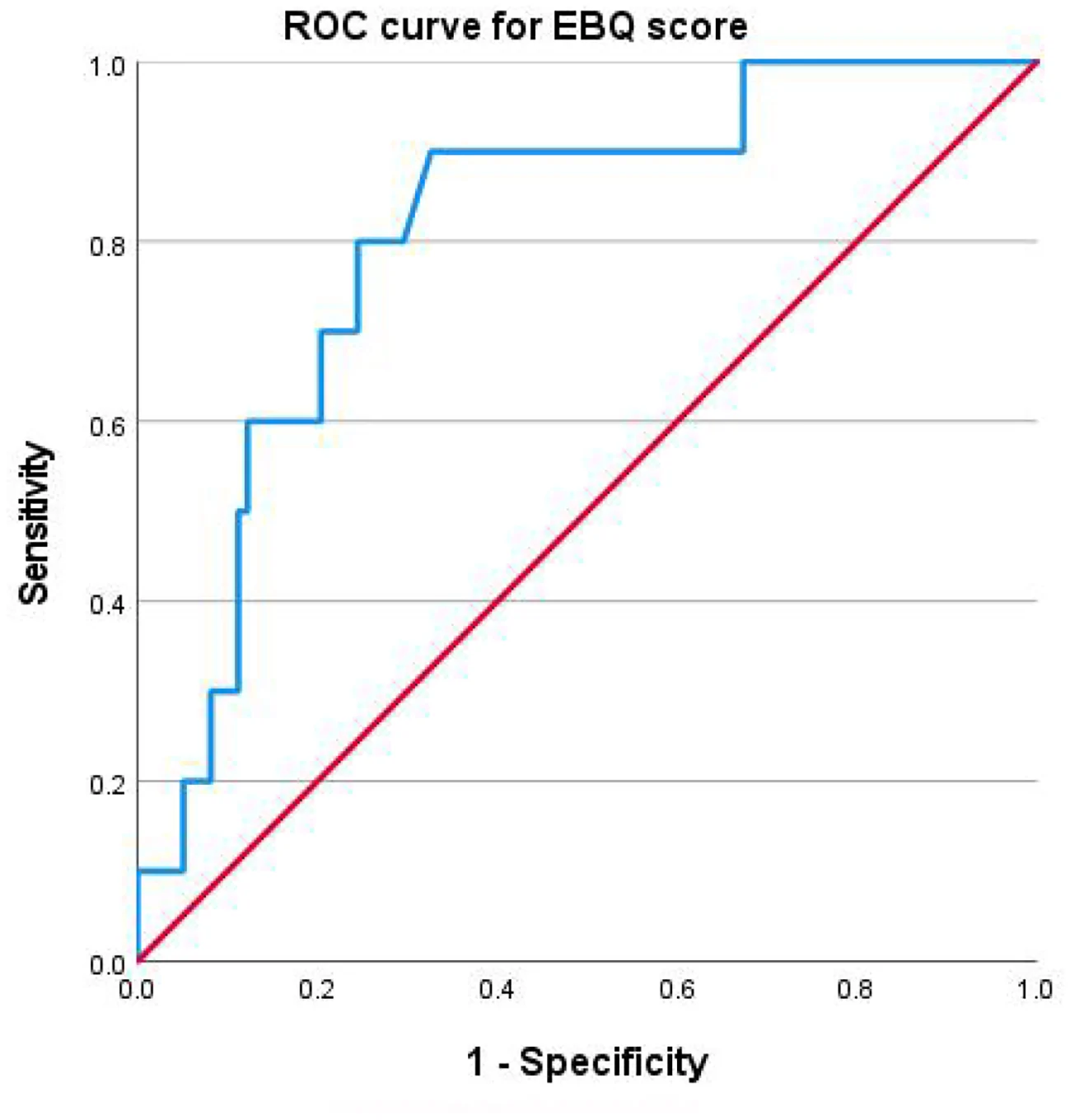
ROC curve analysis and AUC of the EBQ score for the prediction of cage subsidence.

In the sclerotic group, a lower score was significantly associated with an increased risk of postoperative interbody non-fusion [odds ratio (OR) = 1.404; 95% confidence interval (CI): 1.007-1.956; P < 0.05]. On the ROC curve, the AUC for the EBQ score was 0.784 (95% CI: 0.675-0.892). The optimal EBQ score cutoff was 1.745, yielding a sensitivity of 72.6% and a specificity of 78.9% (**Figure 4**).

**Figure 4:**
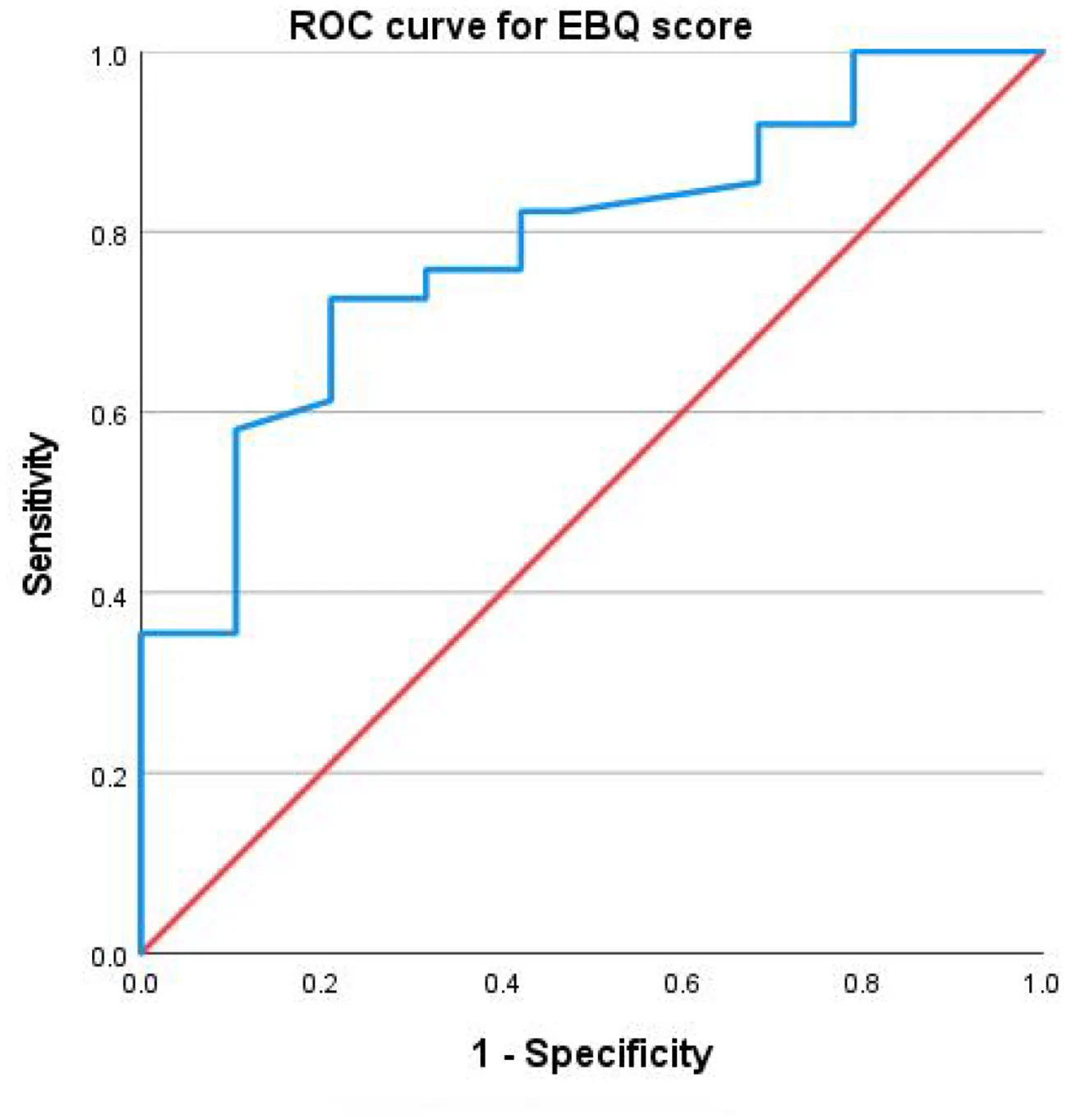
ROC curve analysis and AUC of the EBQ score for the prediction of bony fusion.

## Discussion

This study elucidates a critical dichotomy in postoperative outcomes following PLIF for patients with MCs, contingent upon preoperative endplate morphology. The principal findings demonstrate that the presence of endplate sclerosis is associated with a significantly reduced rate of bony fusion, whereas its absence correlates with an elevated risk of cage subsidence.

Importantly, EBQ showed good discriminatory ability on ROC analysis for both outcomes and demonstrated significant independent associations in multivariable regression models. Nevertheless, the limited events- per- variable ratio (approximately 1.25 for the non- sclerotic group and 2.4 for the sclerotic group with 8 covariates) warrants cautious interpretation of these regression estimates, and validation in larger cohorts is needed.

Our results affirm and substantially extend the established role of EBQ as a surrogate for regional bone density. Prior literature has validated EBQ’s utility in predicting implant subsidence in TLIF procedures (Ai et al., 2024). This study corroborates that predictive value in the non-sclerosis cohort, where diminished bone density logically compromises load-bearing capacity, leading to vertebral body micro-fractures and cage settling. The novel and significant contribution lies in establishing EBQ as an equally powerful predictor for fusion failure in the sclerosis group. This finding challenges a simplistic view of bone quality, indicating that a high-density, sclerotic endplate does not equate to a superior fusion substrate. We hypothesize that this high- density, sclerotic bone, despite its radiographic density, may be biologically and biomechanically inferior for fusion. This aligns with the concept that sclerosis may represent a hypovascular, metabolically quiescent bone state, impairing the migration of osteoprogenitor cells and growth factors from the vertebral body into the interbody graft, ultimately hindering osseous integration (Xiao et al., 2023; Zeng et al., 2023; Fan et al., 2024).However, this mechanistic interpretation was not directly tested in our study and should be investigated in future histologic and molecular studies.

The divergent complication pathways—fusion failure versus mechanical subsidence—highlight the necessity for preoperative risk stratification based on endplate status. Our data support a paradigm where the preoperative assessment should move beyond a global “osteoporosis risk” evaluation to a more nuanced, pathology-specific “endplate quality” assessment. For patients with non-sclerotic endplates and high EBQ, the surgical strategy should prioritize mitigating subsidence risk. This may involve the use of wider-footprint cages, enhanced fixation techniques (e.g., supplemental posterior instrumentation), or potentially stronger graft materials (Goh et al., 2000; Schiffman et al., 2003). Conversely, for patients with sclerotic endplates and low EBQ, the focus must shift to overcoming the biological impediment to fusion. Strategies could include more aggressive endplate decortication to penetrate the sclerotic barrier, the use of osteoinductive bone graft substitutes or biologics (e.g., bone morphogenetic proteins, concentrated bone marrow aspirate), and ensuring optimal graft-host contact (Ji et al., 2020; Li et al., 2021).

The calculated EBQ threshold provides a potential quantitative tool for clinical decision-making. This value, derived from the Youden index, offers an objective cutoff to identify high-risk patients within each subgroup preoperatively. Its high sensitivity and specificity, as evidenced by the AUC, suggest good discriminatory power. Future prospective studies are warranted to validate this threshold in independent cohorts and to test whether interventions tailored to this risk stratification improve clinical outcomes.

Several limitations of this study must be acknowledged. First, its retrospective, single-center design may introduce selection bias and limit generalizability. Second, the sample size, particularly when subdivided by MC types and sclerosis status, may constrain the statistical power for more nuanced analyses. Specifically, the low events- per- variable ratios (1.25 and 2.4 for the two models) increase the risk of overfitting and may inflate the regression coefficients and significance levels; hence, our findings require external validation. Third, while standardized criteria were used, the assessment of fusion and subsidence on radiographic imaging has inherent subjectivity; future studies could benefit from more advanced quantitative CT analysis. Fourth, the EBQ cutoff values were derived using the Youden index from the same dataset that was used for model development, without internal cross- validation or external validation in an independent cohort. This approach is known to produce optimistic estimates of AUC, sensitivity, and specificity (optimism bias). Therefore, the reported cutoffs require validation in future prospective studies before they can be recommended for clinical use.

## Conclusion

In conclusion, our findings suggest that endplate morphology (sclerotic vs. non- sclerotic) may define distinct risk profiles for complications after PLIF in MC patients. The EBQ score shows promise as a predictive marker for both complications, potentially underscoring its dual role in assessing mechanical strength in soft bone and biological receptivity in hard bone.

These preliminary observations advocate for the integration of preoperative endplate quality assessment into surgical planning as a hypothesis- generating strategy, but they require confirmation in future prospective, multi- center research with larger sample sizes to validate our proposed EBQ threshold and to evaluate the efficacy of complication- specific surgical strategies.

## Data Availability

The raw data supporting the conclusions of this article will be made available by the authors, without undue reservation.
